# Comprehensive identification of age-related lipidome changes in rat amygdala during normal aging

**DOI:** 10.1371/journal.pone.0180675

**Published:** 2017-07-03

**Authors:** Roman Šmidák, Harald C. Köfeler, Harald Hoeger, Gert Lubec

**Affiliations:** 1 Department of Pharmaceutical Chemistry, Faculty of Life Sciences, University of Vienna, Vienna, Austria; 2 Center for Medical Research (ZMF), Medical University Graz, Graz, Austria; 3 Core Unit of Biomedical Research, Division of Laboratory Animal Science and Genetics, Medical University of Vienna, Himberg, Austria; 4 Neuroproteomics Laboratory, Science Park, Ilkovicova 8, Bratislava, Slovakia; Max Delbruck Centrum fur Molekulare Medizin Berlin Buch, GERMANY

## Abstract

Brain lipids are integral components of brain structure and function. However, only recent advancements of chromatographic techniques together with mass spectrometry allow comprehensive identification of lipid species in complex brain tissue. Lipid composition varies between the individual areas and the majority of previous reports was focusing on individual lipids rather than a lipidome. Herein, a mass spectrometry-based approach was used to evaluate age-related changes in the lipidome of the rat amygdala obtained from young (3 months) and old (20 months) males of the Sprague-Dawley rat strain. A total number of 70 lipid species with significantly changed levels between the two animal groups were identified spanning four main lipid classes, i.e. glycerolipids, glycerophospholipids, sphingolipids and sterol lipids. These included phospholipids with pleiotropic brain function, such as derivatives of phosphatidylcholine, phosphatidylserine, and phosphatidylethanolamine. The analysis also revealed significant level changes of phosphatidic acid, diacylglycerol, sphingomyelin and ceramide that directly represent lipid signaling and affect amygdala neuronal activity. The amygdala is a crucial brain region for cognitive functions and former studies on rats and humans showed that this region changes its activity during normal aging. As the information on amygdala lipidome is very limited the results obtained in the present study represent a significant novelty and may contribute to further studies on the role of lipid molecules in age-associated changes of amygdala function.

## Introduction

Brain lipids are attracting focused attention in neurochemistry as information carrying molecules that affect the biological processes by both, modifying membrane structures and direct interaction with other signaling molecules [1,2]. The numerous functions of lipids reflect their high structural and combinatorial diversity that makes lipidomic analyses experimentally challenging [3]. These analyses have been frequently hampered by sampling problem, extraction procedures as well as instability in postmortem tissues [4]. However, the advancements in chromatographic separations and mass spectrometry (MS) currently allow more complex identification and quantitation of lipid species in different types of biological samples [5].

Bozek et al. [6] recently used an MS-based non-targeted approach to comprehensively characterize the tissue-specific lipid composition in different mammalian models. Based on analysis of over 5,000 hydrophobic compounds the authors showed that the brain was significantly different as compared to other non-neural tissues and highlighted the specific role of neural lipids. A series of other studies revealed brain lipid changes in association with Alzheimer’s disease (AD) [7,8], Parkinson’s disease [9,10] or schizophrenia [11,12] and supported the relevance of lipidomic analyses of model organisms for studying neurological disorders [13,14].

Information about lipidome changes in the aged rat brain is limited. Moreover, brain aging occurs in a region-specific manner [15] and lipid profiles in the individual regions of the brain are intrinsically different [2,16]. This further adds to the complexities of the interpretation of lipidomic data. Previous studies on rat brain showed aging-induced alterations of individual lipid species in the whole brain or selected brain areas [17–21]. However, the majority of these analyses lack either comprehensive lipid annotation or spatial resolution of the analyzed brain tissue.

Recently, a comprehensive lipidome of rat amygdala has been analyzed with respect to chronic stress conditions [22]. In the current study, a comprehensive MS-based approach to identify age-associated changes in the lipidome of the amygdala from Sprague-Dawley rats was attempted. The amygdala, a brain region crucial for the formation of emotional memory, modulates fear, anxiety, motivation and social behaviour [23, 24]. Previous studies on rats showed that age impairs amygdala-dependent synaptic plasticity [25] and induced emotion-related behavioural changes associated with reduced neuronal activity in this region [26]. However, the molecular basis of age-related changes in the amygdala is still unknown. Our results provides the list of a total number of 70 lipid species with pleiotropic brain functions and/or more directly involved in cell signaling that showed significantly changed levels in the rat amygdala during aging.

## Materials and methods

### Animals

Male Sprague-Dawley rats were bred and maintained in the animal facility Core Unit of Biomedical Research, Division of Laboratory Animal Science and Genetics, Medical University of Vienna, Himberg, Austria. The animals were maintained in cages made of Makrolon filled with autoclaved woodchips. The conditions were as follows: room temperature 22 ± 1°C, relative humidity 50 ± 10%, light/dark rhythm 14:10 h. Ventilation with 100% fresh air resulted in an air change rate of 15 times per hour. Two groups (12 animals per group) of Sprague-Dawley rats, 3 months old with an average weight of 440 g and 20 months old with an average weight of 650 g were used in this study. All procedures were carried out according to the guidelines of the European Communities Council Directive of 24 November 1986 (86/609/EEC), evaluated by the ethics committee of the Medical University of Vienna, Vienna, Austria and were approved by Federal Ministry of Education, Science and Culture, Austria. The animals were anesthetized by intraperitoneal injection of 50 mg/kg sodium pentobarbital and sacrificed by decapitation. The amygdala was extracted following a micro-dissection procedure as previously described [27] and frozen in -80°C for lipidomic analysis.

### Analysis of lipids

Lipids were extracted by a methyl-tert-butyl ether (MTBE) protocol as previously described [28] with 2.5 nmol PC 12:0/12:0 (Avanti Polar Lipids, Alabaster, AL, USA) added to each sample before extraction for monitoring the extraction efficiency. Briefly, pieces of rat brain were put in 4 mL of methanol / MTBE 1:1.67 and homogenized on ice for 30s by an Ultra-Turrax homogenizer. After addition of 2.5 mL MTBE and 1.25 mL aqua bidest the upper phase was taken off and the lower phase was re-extracted with another 2mL MTBE. The combined organic phases were dried and resuspended in 1000μL methanol / chloroform 1:1 and an aliquot thereof was again dried and resuspended in 100μL isopropanol:chloroform:methanol (90:5:5 v/v/v). Data acquisition was performed on an LTQ Orbitrap Velos Pro instrument (Thermo Scientific) coupled to a Dionex Ultimate 3000 UHPLC (Thermo Scientific) according to previously published protocols [29, 30]. In a nutshell, chromatographic separation was performed on a Waters (Waters, Milford, MA, USA) BEH C8 column (100 × 1 mm, 1.7 μm), thermostatted to 50°C. The mobile phase A was deionized water containing 1 vol% of 1M aqueous ammonium formate (final concentration 10 mmol/L) and 0.1 vol% of formic acid as additives. The mobile Phase B was a mixture of acetonitrile/isopropanol 5:2 (v/v) with the same additives. Gradient elution started at 50% mobile phase B, rising to 100% B over 40 minutes; 100% B were held for 10 minutes and the column was re-equilibrated with 50% B for 8 minutes before the next injection. The flow rate was 150 μL/min, the samples were kept at 8°C and the injection volume was 2 μL. The mass spectrometer was operated in Data Dependent Acquisition mode using an HESI II ion source. Every sample was measured once in positive polarity and once in negative polarity. Ion source parameters for positive polarity were as follows: Source Voltage: 4.5 kV; Source Temperature: 275°C; Sheath Gas: 25 arbitrary units; Aux Gas: 9 arbitrary units; Sweep Gas: 0 arbitrary units; Capillary Temperature: 300°C. Ion source parameters for negative ion mode were: Source Voltage: 3.8 kV; Source Temperature: 325°C; Sheath Gas: 30 arbitrary units; Aux Gas: 10 arbitrary units; Sweep Gas: 0 arbitrary units; Capillary Temperature: 300°C. Automatic gain control target value was set to 106 ions to enter the mass analyzer, with a maximum ion accumulation time of 500 ms. Full scan profile spectra from m/z 350–1500 for positive ion mode and from 350–1600 in negative ion mode were acquired in the Orbitrap mass analyzer at a resolution setting of 100 000 at m/z 400. For MS/MS experiments, the 10 most abundant ions of the full scan spectrum were sequentially fragmented in the ion trap using He as collision gas (CID, Normalized Collision Energy: 50; Isolation Width: 1.5; Activation Q: 0.2; Activation Time: 10) and centroided product spectra at normal scan rate (33 kDa/s) were collected. The exclusion time was set to 10 s.

### Data analysis

Differential lipidomic analysis of young versus aged animals was performed by SIEVE™ software (version 1.3, Thermo Scientific). Briefly, peaks were detected and aligned into features (m/z width: 10 ppm; RT width: 2.5 min) by SIEVE™ software with subsequent manual feature identification of all significantly up- or downregulated species (P-Value < 0.05). Annotation of lipid species is according to the Lipid MAPS shorthand nomenclature [31]. The peak areas corresponding to individual molecular species are presented as ratio values between young and old animals of peak areas averaged across the samples within one group ± SEM. Lipid species are denoted as AA X:Y based on the abbreviation of lipid molecule (AA), the total number of carbons (X) and the total number of double bonds (Y) in their acyl side chains.

## Results

The aim of this study was to perform a high throughput non-targeted lipidomic analysis to identify significantly changed lipid species in the amygdala of the aged rat brain. The dissected amygdalae of young (3 months) and old (20 months) animals of Sprague-Dawley rats, each group containing 12 individuals were analyzed by liquid chromatography coupled to electrospray ionization mass spectrometry (ESI-MS). To achieve a comprehensive detection, the MS experiment was run in both, negative and positive ionization mode. Significantly changed lipid species were identified by accurate (+/- 5 ppm) precursor mass matching with an internal lipid database which covers more than 20,000 molecular lipid species originating from 58 individual lipid (sub)classes. All identified lipid molecules, the polarity of the molecules, fold change and P-values are summarized in **Table 1** and more detailed experimental data are provided as supporting information in **S1 Table**.

**Table 1:** The summary of identified lipid species with significantly changed levels in the amygdalae of aged rats in compare to young individuals.

Lipid MAPS classification	Lipid Molecule	Polarity of molecule	Log2 Ratio (Aged vs. Young)	SEM	P-Value
Glycerophosphocholines	**aPC 32:3**	+	**1.02**	0.25	0.008
	**aPC 34:2**	+	**1.20**	0.28	0.005
	**aPC 34:3**	+	**1.74**	0.38	0.013
	**aPC 36:0**	+	**-1.17**	0.26	0.001
	**aPC 36:1**	+	**-1.61**	0.21	0.000
	**aPC 36:2**	+	**-1.61**	0.43	0.014
	**aPC 38:1**	+	**-1.04**	0.31	0.011
	**aPC 38:2**	+	**-1.35**	0.37	0.012
	**aPC 38:2**	+	**-1.37**	0.84	0.033
	**aPC 40:2**	+	**-1.10**	0.22	0.004
	**LPC 20:4**	+	**-1.31**	0.25	0.000
	**LPC 22:6**	+	**-1.27**	0.25	0.000
	**PC 32:0**	+	**-1.25**	0.47	0.050
	**PC 32:2**	+	**-1.46**	0.28	0.000
	**PC 36:1**	+	**-1.49**	0.57	0.014
	**PC 40:0**	+	**-1.25**	0.24	0.001
	**PC 42:3**	+	**-1.05**	0.39	0.048
	**PC 42:4**	+	**-1.18**	0.29	0.001
	**PC 42:5**	+	**-1.16**	0.27	0.001
	**PC 42:8**	+	**-1.03**	0.29	0.007
	**PC 44:1**	+	**-1.12**	0.28	0.003
	**PC 44:2**	+	**-1.11**	0.27	0.002
	**PC 44:8**	+	**-1.39**	0.26	0.000
	**PC 48:5**	+	**-1.67**	0.39	0.006
	**PC 40:3**	-	**-2.69**	0.22	0.001
Glycerophosphoethanolamines	**aPE 38:1**	+	**-1.33**	0.26	0.004
	**aPE 38:6**	+	**-1.11**	0.20	0.000
	**aPE 40:7**	+	**-1.86**	0.41	0.005
	**aPE 42:5**	+	**-1.01**	0.24	0.001
	**LPE 20:4**	+	**-1.16**	0.23	0.000
	**LPE 22:6**	+	**-1.09**	0.21	0.000
	**PE 38:0**	+	**-1.08**	0.28	0.003
	**PE 38:4**	+	**-1.02**	0.20	0.001
	**PE 40:5**	+	**-1.08**	0.16	0.000
	**PE 42:4**	+	**-1.07**	0.22	0.001
	**PE 42:5**	+	**-1.31**	0.24	0.000
	**PE 42:8**	+	**-1.04**	0.26	0.002
	**PE 44:8**	-	**-1.11**	0.15	0.000
Glycerophopshoserines	**PS 46:2**	-	**-2.38**	0.25	0.000
Glycerophosphoglycerophosphoglycerols (Cardiolipins)	**CL 82:9**	-	**-1.46**	0.20	0.000
Diacylglycerophosphates	**PA 34:1**	-	**1.17**	0.15	0.000
(Phosphatidic acid)	**PA 36:1**	-	**1.19**	0.15	0.000
Diacylglycerols	**DG 32:0**	+	**-1.32**	0.25	0.000
	**DG 40:6**	+	**-1.26**	0.32	0.007
	**DG 42:5**	+	**-1.62**	0.31	0.002
Triacylglycerols	**TG 46:0**	+	**-0.99**	0.19	0.002
	**TG 46:1**	+	**-1.53**	0.27	0.004
	**TG 48:0**	+	**-1.03**	0.24	0.006
	**TG 48:1**	+	**-1.46**	0.25	0.000
	**TG 48:2**	+	**-1.30**	0.35	0.002
	**TG 54:7**	+	**-1.13**	0.36	0.013
	**TG 58:10**	+	**-0.91**	0.35	0.030
	**TG 58:9**	+	**-1.06**	0.37	0.019
	**TG 60:11**	+	**-1.11**	0.38	0.018
Cholesterol esters	**CE 20:4**	+	**1.60**	0.37	0.002
Ceramides	**Cer 20:0**	+	**-1.04**	0.27	0.003
	**Cer 36:1 [O]**	+	**-1.28**	0.31	0.004
Dihydroceramides	**DHCer 18:0**	+	**-1.21**	0.25	0.001
	**DHCer 24:0 [O]**	+	**-2.08**	0.27	0.000
Hexosyceramides	**HexCer 20:1**	+	**1.31**	0.15	0.000
	**HexCer 26:1**	+	**-1.19**	0.29	0.002
	**HexCer 28:3**	+	**-1.05**	0.18	0.000
Hexosyldihydroceramides	**HexDHCer 22:0**	+	**-3.25**	0.35	0.000
	**HexDHCer 24:0**	-	**-2.58**	0.25	0.000
Sphingomyelins	**SM 22:0**	-	**1.01**	0.14	0.000
	**SM 22:1**	-	**1.11**	0.22	0.001
	**SM 24:0**	-	**1.22**	0.17	0.000
	**SM 24:1**	-	**1.57**	0.19	0.000

Lipid species are denoted as AA X:Y based on the abbreviation of lipid molecule (AA), the total number of carbons (X) and the total number of double bonds (Y) in their acyl side chains. The values are represented as base 2 logarithm of the aged vs. young ratio of averaged peak areas ± SEM. P-Values were calculated based on SIEVE™ software (Thermo Scientific). The lipid molecules are assigned to lipid classes according to Lipid MAPS classification (http://www.lipidmaps.org/) system. The abbreviations of lipid molecules are: **aPC** alkyl-/acyllglycerophosphocholine (alkyl-/acylphosphatidylcholine), **aPE** alkyl-/acylglycerophosphoethanolamine (alkyl-/acylphosphatidylethanolamine), **CE** cholesterol ester, **Cer** ceramide, **CL** glycerophosphoglycerophosphoglycerol (Cardiolipin), **DG** diacylglycerol, **DHCer** dihydroceramide, **HexCer** hexosylceramide, **HexDHCer** hexosyldihydroceramide, **LPC** lysoglycerophosphocholine (lysophosphatidylcholine), **LPE** lysoglycerophosphoethanolamine (lysphosphatidylethanolamine), **PA** phosphatidic acid, **PC** diacylglycerophosphocholine (phosphatidylcholine), **PE** diacylglycerophosphoethanolamine (phosphatidylethanolamine), **PS** diacylglycerophosphoserine (phosphatidylserine), **SM** sphingomyelin, **TG** triacylglycerol

In our analysis, 70 lipid species have been detected with significantly changed levels (P-value < 0.05) between two groups. Based on the Lipid MAPS classification system (http://www.lipidmaps.org/) the identified lipid molecules have been assigned to four main lipid classes: glycerolipids, glycerophospholipids, sphingolipids and sterol lipids spanning ten distinct lipid subclasses: di- and triacylglycerols, glycerophosphates, glycerophospho- / -cholines / -ethanolamines / -serines, glycerophosphoglycerophosphoglycerols (cardiolipins), ceramides, sphingomyelins and sterols.

**Fig 1:**
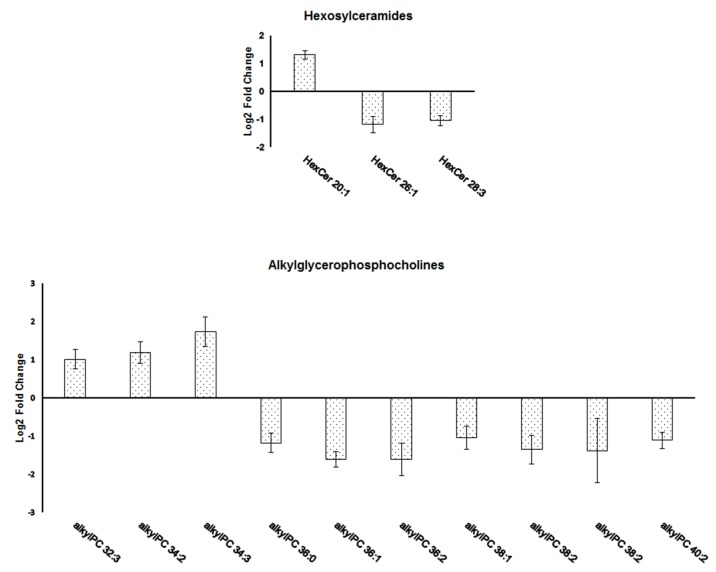
Level changes of the molecular species of hexosylceramides (HexCer) and alkyl-/acylglycerophospholipids (aPC) significantly dysregulated in the amygdalae of aged vs. young rats. Individual lipid molecules were differentially regulated based on length and saturation of fatty acyl chains. Lipid species are denoted as AA X:Y based on the abbreviation of lipid molecule (AA), the total number of carbons (X) and the total number of double bonds (Y) in their acyl side chains. The values are represented as base 2 logarithm of the aged vs. young ratio of averaged peak areas ± SEM.

Most of the identified lipid species showed significantly decreased levels in the group of aged rats. These include diacylglycerols (DG) and triacylglycerols (TG); diacylglycerophosphocholines (PC) and lysoglycerophosphocholines (LPC); identified derivatives of glycerophosphoethanolamines and glycerophosphoserines; ceramides (Cer), hexosyldihydroceramides (HexDHCer) and dihydroceramides (DHCer). The opposite trend was observed for seven members of cholesterol esters (CE), sphingomyelins (SM) and diacylglycerophosphates (or phosphatidic acid, PA) that were significantly upregulated in the group of aged animals. While all of these lipids showed the same direction of fold change within the same lipid group our analysis also detected classes with differential regulation of its individual members: hexosylceramides (HexCer) and alkyl-/acylglycerophosphocholines (aPC) (**Fig 1**). For aPCs, the data showed a significant increase of members containing shorter polyunsaturated chains (aPC 32(34):2(3)) with a simultaneous decrease in longer and more saturated chains (aPC 36(38,40):0(1,2,3)) in aged animals. A similar effect was observed for HexCers with an increase of Cer 20:1 species and a decrease of HexCer 26:1 and HexCer 28:3 in the group of aged rats.

## Discussion

Lipids form a substantial part of the brain and are pleiotropic in function. So far, very few studies analyzed the impact of aging on distinct areas in rat brain using lipidomic approach. Although amygdala is one of the primary brain structures responsible for the formation of memory and controlling emotional behaviour, lipidomic data for this area is very limited. In the current study, a comprehensive lipidomic analysis of rat amygdala was performed to better understand the age-related changes of lipid signaling in this region.

Significant changes were detected for several classes of glycerophospholipids with a decrease of all identified diacylglycerophosphocholines (PC), lysoglycerophosphocholines (lysoPC), glycerophosphoserines and glycerophosphoethanolamines, and an increase of phosphatidic acid (PA) in the amygdala of aged rats. Similar trends during normal aging were shown previously for PC, glycerophosphoethanolamines, diacylglycerophoshoserines and PA in mouse brain [32], and for glycerophosphoethanolamines in the frontal cortex of rat brain [19]. This consistency suggests certain homogeneity across different brain regions and is in line with more general functions of these lipids in the nervous system. Glycerophosphocholines, glycerophosphoethanolamines and glycerophosphoserines play a major role in neuronal differentiation and morphogenesis [33,34] and their alteration may directly affect the action of many signaling molecules. As an example, diacylglycerophosphoserine (phosphatidylserine, PS) is recognized by different soluble proteins including kinases Akt and Raf-1 and protein kinase C [35–37], and PC is the main precursor for the synthesis of two important lipid second messengers, PA and diacylglycerol (DG) [1]. Interestingly, PS also modulates glutamate receptor function [38] associated with the role of the amygdala in fear memory [39].

**Fig 2:**
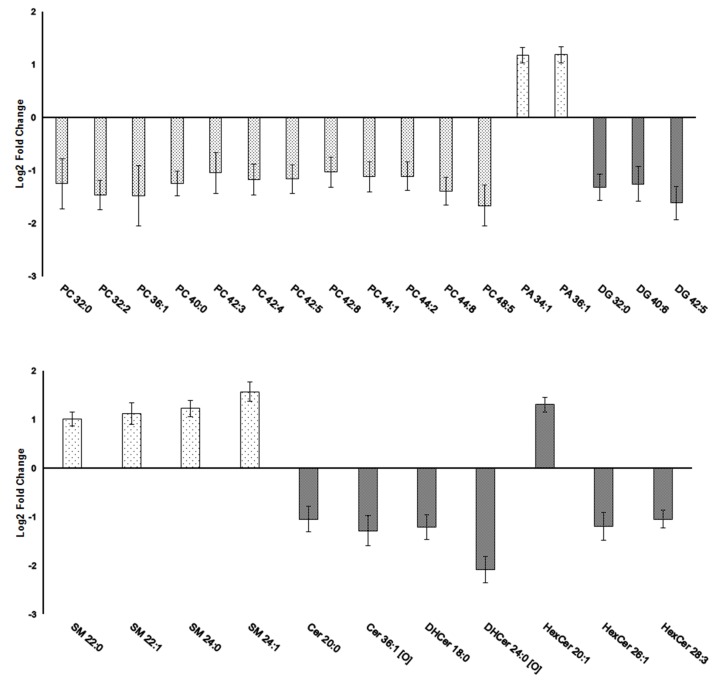
Level changes of important signaling lipids dysregulated in the amygdalae of aged vs. young rats. Several molecules of glycerophospholipids (first graph): phosphatidic acid (PA), diacylglycerols (DG) and glycerophosphocholines (PC), and members of sphingolipids (second graph): sphingomyelins (SM), ceramides (Cer) and its derivatives hexosylceramides, hexosyldihydroceramides (HexDHCer) and dihydroceramides (DHCer) have shown significantly different levels in the amygdalae of aged as compared to young rats. Lipid species are denoted as AA X:Y based on the abbreviation of lipid molecule (AA), the total number of carbons (X) and the total number of double bonds (Y) in their acyl side chains. The values are represented as base 2 logarithm of the old/young ratio of averaged peak areas ± SEM.

Identification of PA and DG represents a direct modification of lipid signaling (**Fig 2**). PA is produced by the conversion of PC through the action of phospholipase D (PLD), it can be further dephosphorylated by phosphatide phosphohydrolase (PAP, lipin) to generate DG [1] which in turn can be converted back to PA by diacylglycerol kinase (DGK) [2]. Both PA and DG are involved in a variety of signaling events: PA is a negatively charged phospholipid that mediates membrane fusion and vesicle trafficking, and interacts with and/or modulates the activity of many memory-related signaling proteins [40–42]. DG activates a number of effector molecules including protein kinases C, D or MAP [2,43]. An increase in levels of PA and a decrease of PC and DG species in amygdalae of aged rats observed in our analysis points to alterations of enzyme activities involved in the biosynthesis of these lipids. In agreement, previous studies have shown that activities of PLD, PAP and DGK are subject to age-related changes in rat cortex resulting in different PA and DG levels during basal conditions or in response to receptor-mediated signaling [44]. PLD activity in the amygdala has also been identified as a key downstream element used by glutamate, dopamine and serotonin receptor signaling in associative learning [45–47]. An additional link to AD was provided by detection of enhanced PLD activity in primary neurons after amyloid β application and further corroborated by genetic ablation of PLD2 gene which rescued long-term potentiation (LTP) and memory impairment in an AD mouse model (swAPP)[48,49]. In response to swAPP overexpression PLD2 selectively upregulated PA species in mouse brain [49] but the correlation of individual PLD, PAP and DGK activities in this case and conditions associated with normal aging remained to be clarified. Altogether, PA and DG play a key role in neuronal signaling and pathology, and results of the present study strongly suggest a link between alteration of their levels and changes in amygdala activity during aging.

Our lipidomic analysis revealed changes of sphingomyelins (SM) and ceramides (Cer) in the amygdala during aging (**Fig 2**). Cer is a lipid second messenger implicated in neuronal differentiation, cellular proliferation and death [50] that is mainly synthetized from SM [51]. Previous studies linked the chronic increase of Cer levels to normal aging of mouse brain as well as to the pathogenesis of AD [52]. Further analysis of mouse transgenic models of AD also suggested a positive correlation between Cer synthesis and toxic amyloid β accumulation and neuronal loss [53]. In contrast, all but one (HexCer 20:1) Cer species identified in our experiment were decreased in the aged amygdala together with elevated levels of SM. A possible explanation for a general decrease of Cer members is increased utilization of Cer in downstream signaling which was reported previously in the aged rat brain [54]. On the other hand, our results can reflect age-related differences in Cer metabolism specific for amygdala. During brain aging, the neurons experience a higher amount of oxidative stress that increases their vulnerability to degeneration [55]. However, the amygdala was shown to preserve the structural integrity and fundamental functionality during aging [56,57]. Consistently, increased SM levels and reduced sphingomyelinase activity leading to decreased Cer production have been related to increased resistance of neurons to oxidative stress [58]. Finally, the association between modulation of Cer and brain aging seems to be more complex and determined by Cer composition as well as by Cer distribution within brain regions and subcellular compartments [50]. Modulation of SM, Cer as well as cholesterol derivatives identified in our study can also have a more specific effect on neuronal activity in amygdala since these lipids are integral components of lipid rafts enriched in G protein-coupled receptors and crucial for monoamine receptor signaling [59,60].

Individual members of the same lipid class can possess a different ability to regulate physiological processes and/or interact with other molecules [61]. In this sense, we have detected differential regulation of identified hexosylceramides (HexCer) and alkyl-/acylglycerophosphocholines (aPC) based on length and saturation of fatty acid chains. Other lipidomic studies have shown differences across glycerophospholipid and sphingolipid classes in response to stress [22], AD pathology and normal aging [32] in rodent brain. However, further and more detailed analysis are needed to unravel the impact of these changes in the context of cellular metabolism, signaling and function of the amygdala.

Age-related changes in the lipidome reported in the present study showed a modification of lipid signaling and may reflect the altered activity of the amygdala. The results also suggest important links to physiological processes and molecules previously recognized in brain aging and/or amygdala functionality. The current analysis thus provided valuable data on age-related molecular changes in rat amygdala that warrants further investigation.

## Supporting information

S1 Table The summary of identified lipid molecules with significantly changed levels in the amygdalae of aged as compared to young rats. The list of identified lipid molecules, experimental parameters, Lipid MAPS classification and statistics.
(XLSX)
